# Supplementary material for: Image-based metric of invasiveness predicts response to adjuvant temozolomide for primary glioblastoma
Source: PLoS One. 2020 Mar 27;15(3):e0230492. doi: 10.1371/journal.pone.0230492 (PMC7100932; doi:10.1371/journal.pone.0230492)
Supplement: S1 Fig — Top row: In the case of no other treatment until later recurrence, the post-adjuvant image is the first image collected following the last cycle of TMZ. Middle row: in the case of co-administered therapy, the post-adjuvant image is the first image collected following the last cycle of TMZ administered alone/before an additional therapy was introduced. Last row: any cycles of TMZ given after an intervening time on another therapy was excluded from our analysis. (DOCX) [file pone.0230492.s001.docx]

**Imaging time points used in relation to therapy timeline**

**
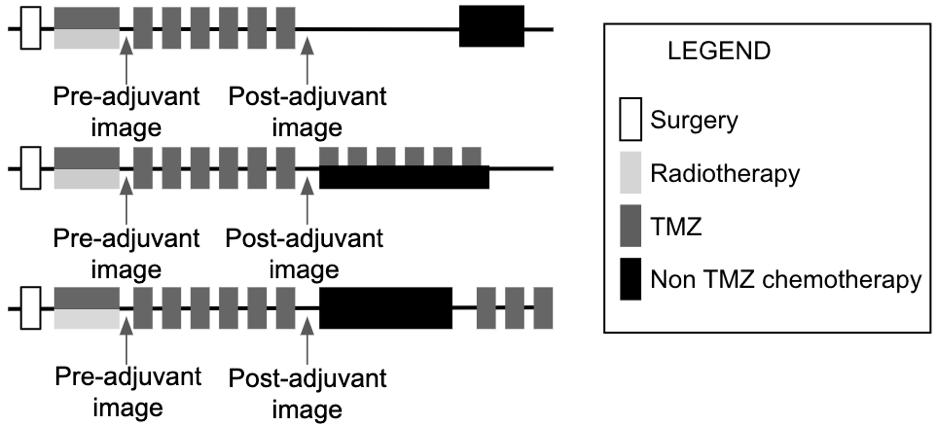
**

**Supplemental Figure S1. Pre- and post-adjuvant imaging time points in relation to treatment time points.** Top row: In the case of no other treatment until later recurrence, the post-adjuvant image is the first image collected following the last cycle of TMZ. Middle row: in the case of co-administered therapy, the post-adjuvant image is the first image collected following the last cycle of TMZ administered alone/before an additional therapy was introduced. Last row: any cycles of TMZ given after an intervening time on another therapy was excluded from our analysis.
