# Supplementary material for: Image-based metric of invasiveness predicts response to adjuvant temozolomide for primary glioblastoma
Source: PLoS One. 2020 Mar 27;15(3):e0230492. doi: 10.1371/journal.pone.0230492 (PMC7100932; doi:10.1371/journal.pone.0230492)
Supplement: S2 Fig — Proportion of adjuvant to nadir is calculated as number of days from pre-adjuvant image to nadir image divided by number of days between pre-adjuvant and post-adjuvant imaging. Top row: Example of tumor reaching nadir volume before post-adjuvant imaging, where %ΔT1Gd-Nadir (about -0.95) is less than %ΔT1Gd (about -0.50). Middle: Example of tumor reaching nadir at post-adjuvant time point, where %ΔT1Gd-Nadir equals %ΔT1Gd. Bottom: Example of how nadir is determined when tumor volume never decreases below pre-adjuvant value. (DOCX) [file pone.0230492.s002.docx]

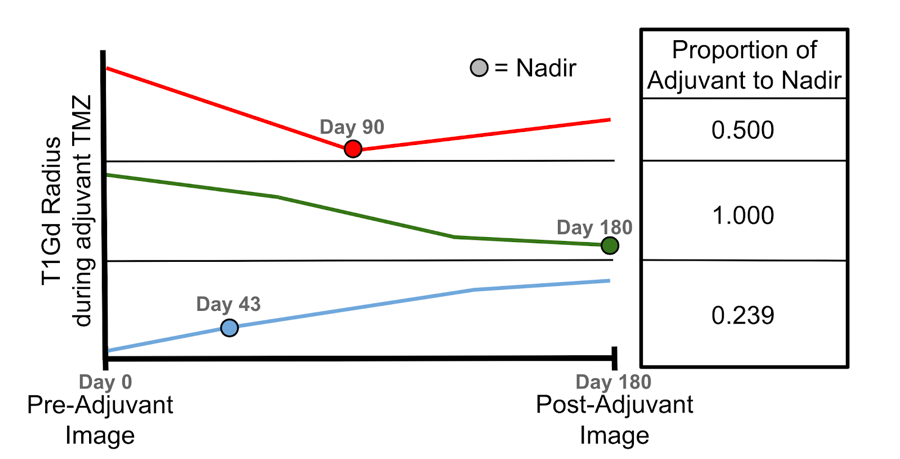


**Supplemental Figure S2. Schematic showing T1Gd radius changes from the pre-adjuvant to post-adjuvant imaging time points to demonstrate how nadir is determined and how proportion of adjuvant to nadir is calculated.** Proportion of adjuvant to nadir is calculated as number of days from pre-adjuvant image to nadir image divided by number of days between pre-adjuvant and post-adjuvant imaging. Top row: Example of tumor reaching nadir volume before post-adjuvant imaging, where %ΔT1Gd-Nadir (about -0.95) is less than %ΔT1Gd (about -0.50). Middle: Example of tumor reaching nadir at post-adjuvant time point, where %ΔT1Gd-Nadir equals %ΔT1Gd. Bottom: Example of how nadir is determined when tumor volume never decreases below pre-adjuvant value.
