# Supplementary material for: Image-based metric of invasiveness predicts response to adjuvant temozolomide for primary glioblastoma
Source: PLoS One. 2020 Mar 27;15(3):e0230492. doi: 10.1371/journal.pone.0230492 (PMC7100932; doi:10.1371/journal.pone.0230492)
Supplement: S3 Fig — The nodular tumors have a negative correlation between number of TMZ cycles received and percent change of T1Gd radius from pre-adjuvant imaging to nadir imaging (F-test, p<0.0001), while the diffuse tumors do not have a significant trend (p = 0.161), supporting the results from Fig 4. (DOCX) [file pone.0230492.s003.docx]

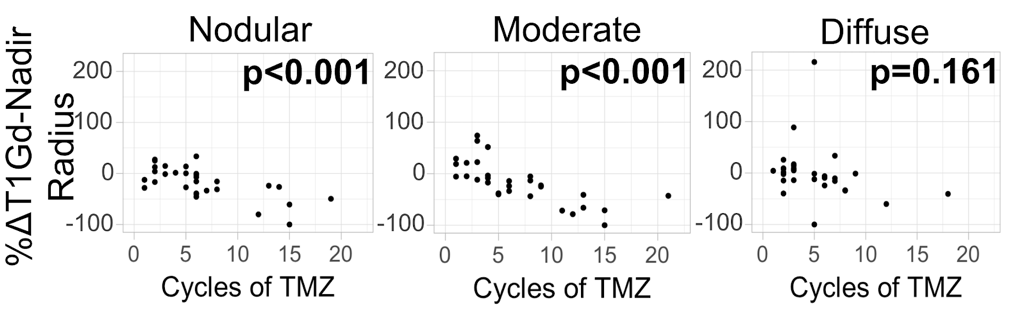


**Supplemental Figure S3. Percent change in T1Gd signal until nadir vs cycles of TMZ.** The nodular tumors have a negative correlation between number of TMZ cycles received and percent change of T1Gd radius from pre-adjuvant imaging to nadir imaging (F-test, p<0.0001), while the diffuse tumors do not have a significant trend (p=0.161), supporting the results from **Figure 4.**
