# Supplementary material for: Image-based metric of invasiveness predicts response to adjuvant temozolomide for primary glioblastoma
Source: PLoS One. 2020 Mar 27;15(3):e0230492. doi: 10.1371/journal.pone.0230492 (PMC7100932; doi:10.1371/journal.pone.0230492)
Supplement: S4 Fig — Although the small sample size limits the statistical power of this comparison, methylated responders tended to have the best survival outcomes compared to other groups, followed by methylated non-responders, unmethylated responders, and unmethylated non-responders. (DOCX) [file pone.0230492.s004.docx]

**MGMT Methylation Status**


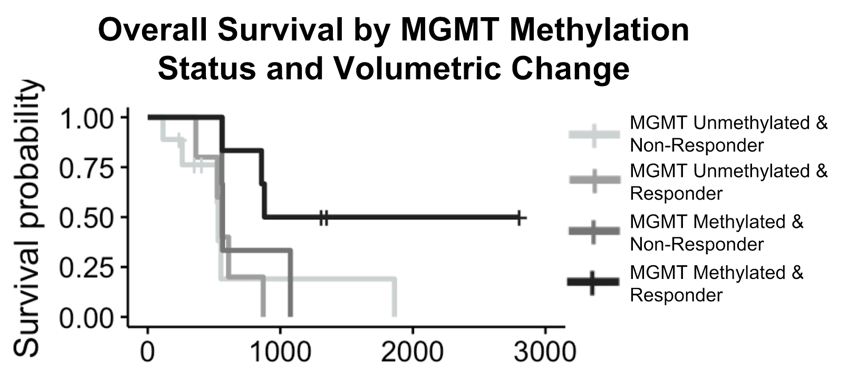


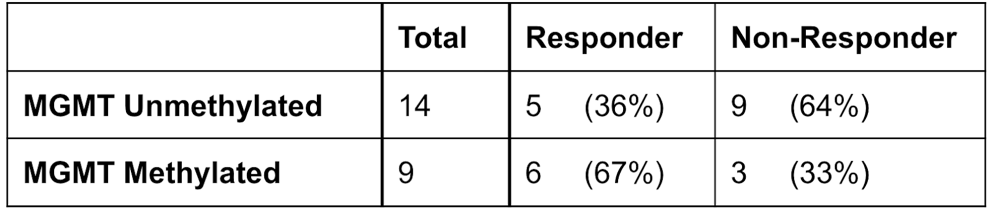


**Supplemental Figure S4. MGMT methylation status and volumetric change for subcohort with methylation status available.** Although the small sample size limits the statistical power of this comparison, methylated responders tended to have the best survival outcomes compared to other groups, followed by methylated non-responders, unmethylated responders, and unmethylated non-responders.
