# Supplementary material for: Image-based metric of invasiveness predicts response to adjuvant temozolomide for primary glioblastoma
Source: PLoS One. 2020 Mar 27;15(3):e0230492. doi: 10.1371/journal.pone.0230492 (PMC7100932; doi:10.1371/journal.pone.0230492)
Supplement: S5 Fig — Among unmethylated tumors, four of the five responders are quite nodular, while the non-responders are distributed more evenly. Methylated patients received significantly more cycles of TMZ than unmethylated patients (t-test p = 0.0156). Among unmethylated patients, responders tended to receive more cycles of TMZ than non-responders. (DOCX) [file pone.0230492.s005.docx]

**
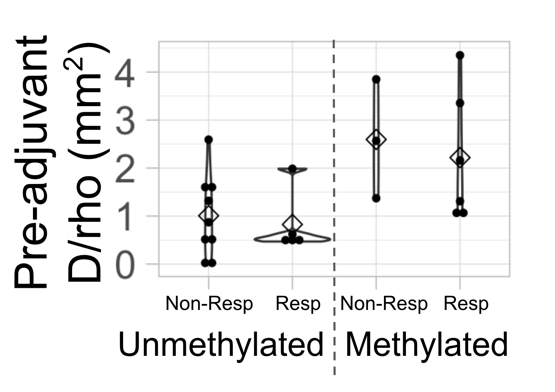

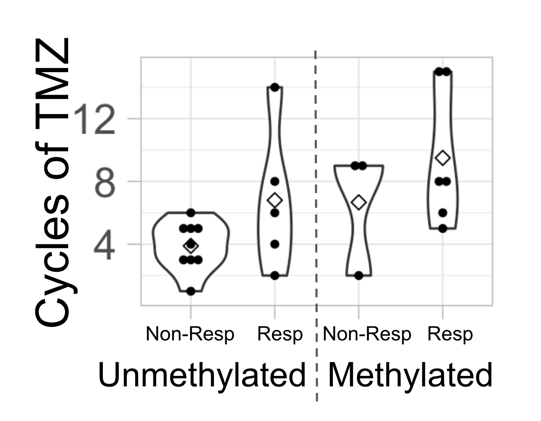
**

**Supplemental Figure S5. Methylated tumors are more diffuse than unmethylated tumors.** Among unmethylated tumors, four of the five responders are quite nodular, while the non-responders are distributed more evenly. Methylated patients received significantly more cycles of TMZ than unmethylated patients (t-test p=0.0156). Among unmethylated patients, responders tended to receive more cycles of TMZ than non-responders.
