# Supplementary material for: Image-based metric of invasiveness predicts response to adjuvant temozolomide for primary glioblastoma
Source: PLoS One. 2020 Mar 27;15(3):e0230492. doi: 10.1371/journal.pone.0230492 (PMC7100932; doi:10.1371/journal.pone.0230492)
Supplement: S6 Fig — This expanded cohort (44 unmethylated, 28 methylated) uses a larger sample size to validate the observation that MGMT methylated tumors are significantly more diffuse than MGMT unmethylated tumors at the pre-adjuvant time point. Therefore, we do not think that the relationship between nodularity and response to TMZ is confounded by a predominance of MGMT methylated tumors in the nodular groups. Patients in this cohort are the 23 MGMT patients from the original patient cohort plus 49 additional patients with available MGMT status and pre-adjuvant D/rho from our database. These additional patients (30 unmethylated and 19 methylated) were first diagnosis GBM patients who received maximal safe resection followed by concurrent radiotherapy (XRT) and chemotherapy (TMZ). Pre-adjuvant d/rho was calculated from the T1Gd and T2-FLAIR images taken after concurrent therapy ended and before adjuvant therapy began. Patients who received therapies other than XRT, TMZ, steroids, or anti-seizure medications before the image was taken were not included in this cohort. (DOCX) [file pone.0230492.s006.docx]

**MGMT Expanded Cohort Validation**


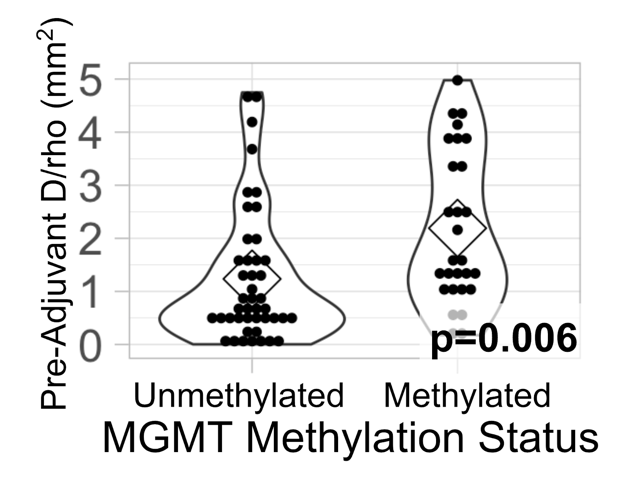


**Supplemental Figure S6. Distribution of pre-adjuvant D/rho vs MGMT methylation status in an expanded cohort.** This expanded cohort (44 unmethylated, 28 methylated) uses a larger sample size to validate the observation that MGMT methylated tumors are significantly more diffuse than MGMT unmethylated tumors at the pre-adjuvant time point. Therefore, we do not think that the relationship between nodularity and response to TMZ is confounded by a predominance of MGMT methylated tumors in the nodular groups. Patients in this cohort are the 23 MGMT patients from the original patient cohort plus 49 additional patients with available MGMT status and pre-adjuvant D/rho from our database. These additional patients (30 unmethylated and 19 methylated) were first diagnosis GBM patients who received maximal safe resection followed by concurrent radiotherapy (XRT) and chemotherapy (TMZ). Pre-adjuvant D/rho was calculated from the T1Gd and T2-FLAIR images taken after concurrent therapy ended and before adjuvant therapy began. Patients who received therapies other than XRT, TMZ, steroids, or anti-seizure medications before the image was taken were not included in this cohort.
