# Supplementary material for: Image-based metric of invasiveness predicts response to adjuvant temozolomide for primary glioblastoma
Source: PLoS One. 2020 Mar 27;15(3):e0230492. doi: 10.1371/journal.pone.0230492 (PMC7100932; doi:10.1371/journal.pone.0230492)
Supplement: S7 Fig — These results are almost the same as those in Fig 1, except the pre-adjuvant D/rho comparison is no longer statistically significant (p = 0.066). (DOCX) [file pone.0230492.s007.docx]

**Pseudoprogression Investigation**

In order to reduce the impact of pseudoprogression on our results, we re-produced figures 1 and 3-5 using all of the patients from the original cohort that had at least 12 weeks between the end of XRT and the post-adjuvant image date (n=72).


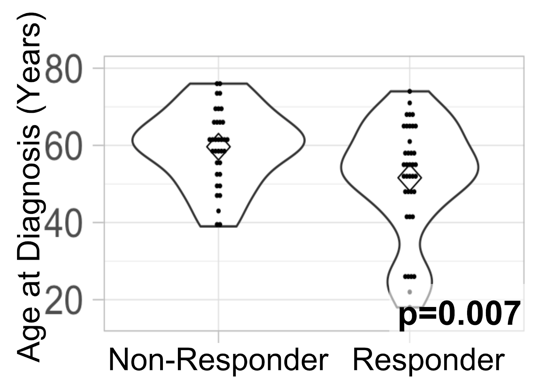

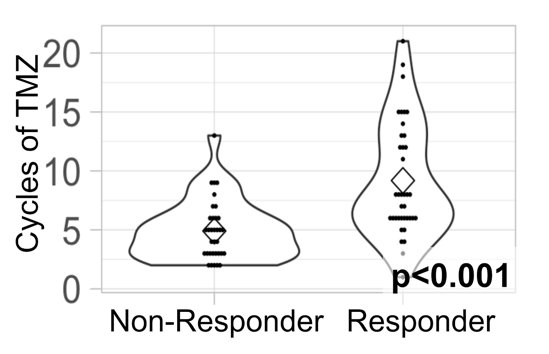


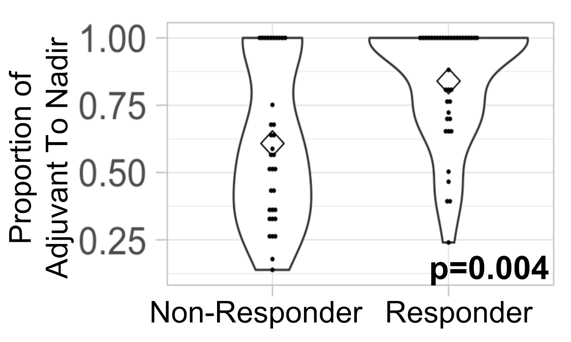

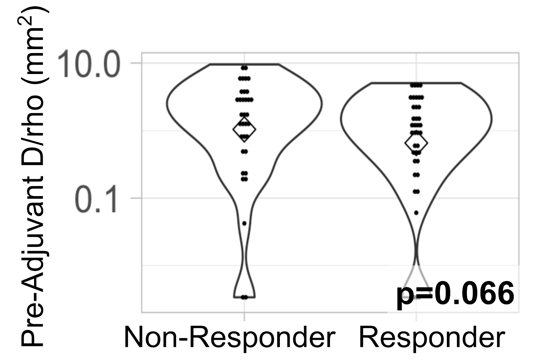


**Supplemental Figure S7. Characteristic differences between responders (n=38) and non-responders (n=34) for subjects with more than 12 weeks between end of XRT and post-adjuvant imaging.** These results are almost the same as those in Figure 1, except the pre-adjuvant D/rho comparison is no longer statistically significant (p=0.066).
