# Supplementary material for: Image-based metric of invasiveness predicts response to adjuvant temozolomide for primary glioblastoma
Source: PLoS One. 2020 Mar 27;15(3):e0230492. doi: 10.1371/journal.pone.0230492 (PMC7100932; doi:10.1371/journal.pone.0230492)
Supplement: S8 Fig — Outcome differences between responders (n = 38) and non-responders (n = 34), show similar results as those in Fig 3. (DOCX) [file pone.0230492.s008.docx]

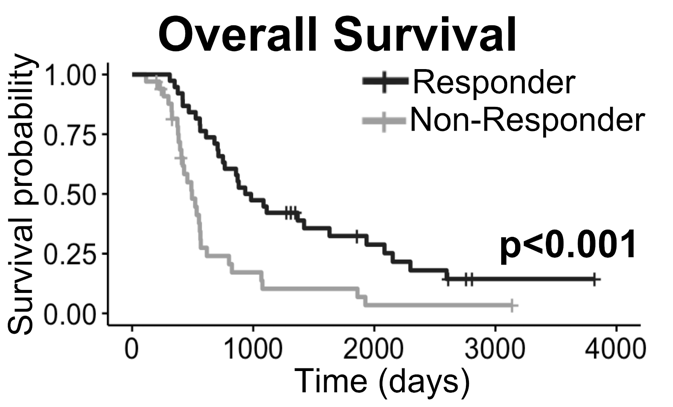

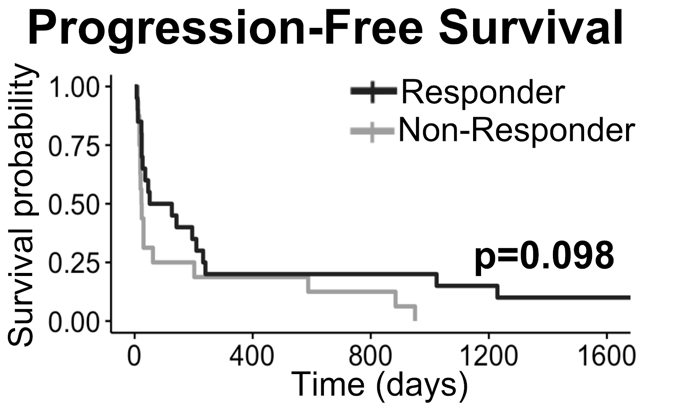


**Supplemental Figure S8. Overall survival and progression-free survival for subcohort with more than 12 weeks between end of XRT and post-adjuvant imaging (n=72).** Outcome differences between responders (n=38) and non-responders (n=34), show similar results as those in Figure 3.
