# Supplementary material for: Image-based metric of invasiveness predicts response to adjuvant temozolomide for primary glioblastoma
Source: PLoS One. 2020 Mar 27;15(3):e0230492. doi: 10.1371/journal.pone.0230492 (PMC7100932; doi:10.1371/journal.pone.0230492)
Supplement: S9 Fig — Similar to the results of Fig 4, nodular tumors (n = 24) show a significant negative correlation between cycles of TMZ received and change in tumor size and this change in tumor size results in a significant survival benefit. Neither the trend nor the survival benefit are observed among diffuse tumors (n = 24). (DOCX) [file pone.0230492.s009.docx]

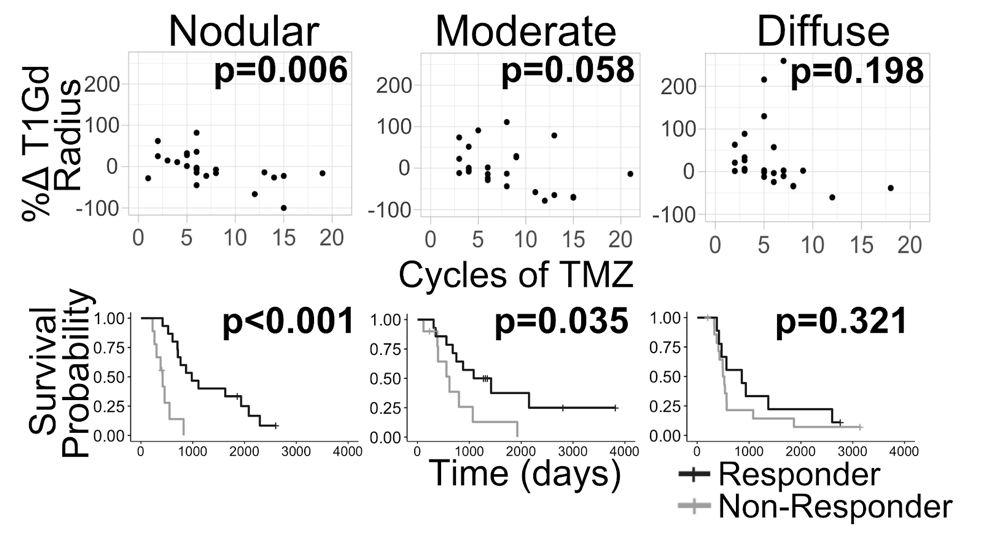


**Supplemental Figure S9. Percent change T1Gd radius and cycles of TMZ as well as survival probabilities for responders and non-responders grouped as nodular, moderate, and diffuse pre-adjuvant tumors for subjects with more than 12 weeks between end of XRT and post-adjuvant imaging (n=72)**. Similar to the results of Figure 4, nodular tumors (n=24) show a significant negative correlation between cycles of TMZ received and change in tumor size and this change in tumor size results in a significant survival benefit. Neither the trend nor the survival benefit are observed among diffuse tumors (n=24).
