# Supplementary material for: Image-based metric of invasiveness predicts response to adjuvant temozolomide for primary glioblastoma
Source: PLoS One. 2020 Mar 27;15(3):e0230492. doi: 10.1371/journal.pone.0230492 (PMC7100932; doi:10.1371/journal.pone.0230492)
Supplement: S10 Fig — Similar to Fig 5, methylated patients (n = 9) show a clearer correlation between cycles of TMZ received and change in tumor size. Unmethylated tumors (n = 10). tend to be more nodular compared to the methylated ones. (DOCX) [file pone.0230492.s010.docx]

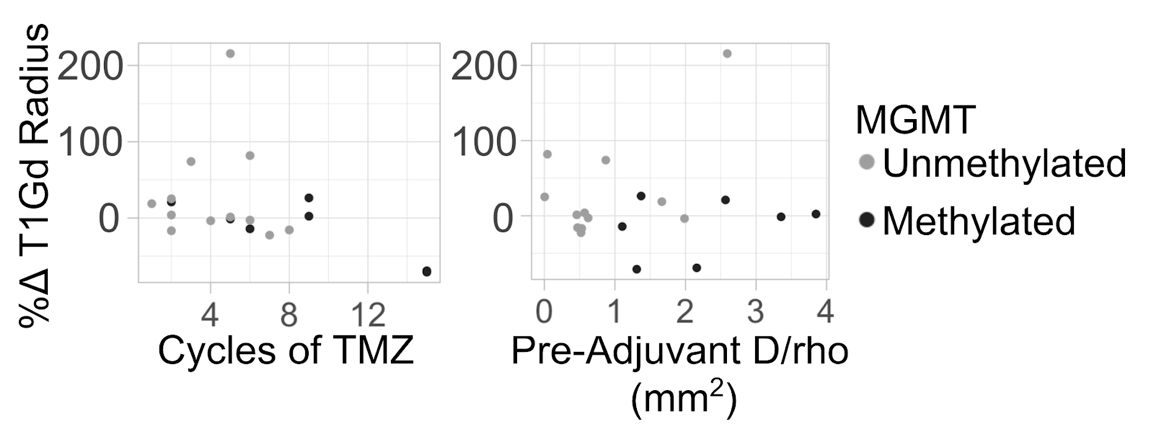


**Supplemental Figure S10. Percent change T1Gd radius vs cycles of TMZ and pre-adjuvant D/rho by MGMT methylation status for subjects with more than 12 weeks between end of XRT and post-adjuvant imaging and methylation status available (n=19).** Similar to Figure 5, methylated patients (n=9) show a clearer correlation between cycles of TMZ received and change in tumor size. Unmethylated tumors (n=10). tend to be more nodular compared to the methylated ones.
