# Supplementary material for: Image-based metric of invasiveness predicts response to adjuvant temozolomide for primary glioblastoma
Source: PLoS One. 2020 Mar 27;15(3):e0230492. doi: 10.1371/journal.pone.0230492 (PMC7100932; doi:10.1371/journal.pone.0230492)
Supplement: S11 Fig — Comparison of characteristics between responders (male n = 28, female n = 17) and non-responders (male n = 32, female n = 13). Males have the same results as the combined population, while the female tests were insignificant. (DOCX) [file pone.0230492.s011.docx]

**Sex differences figures**


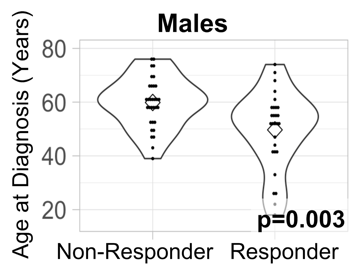

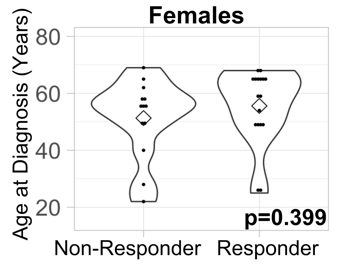

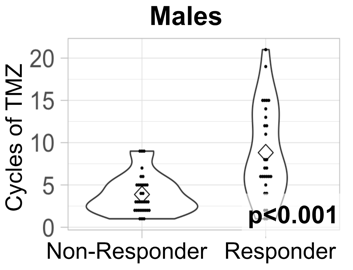

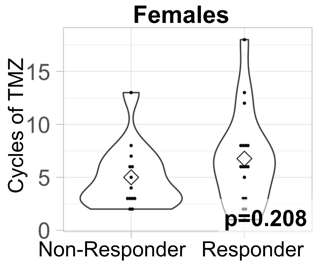


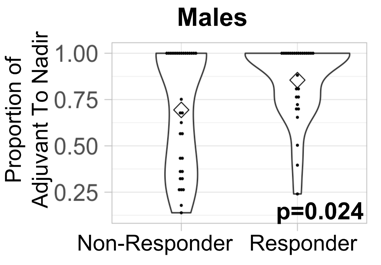

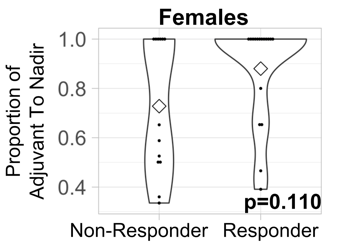

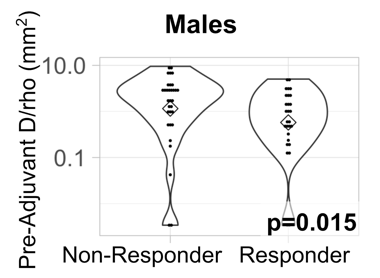

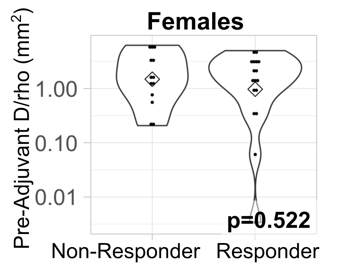


**Supplemental Figure S11. Figure 1 split into males and females.** Comparison of characteristics between responders (male n=28, female n=17) and non-responders (male n=32, female n=13). Males have the same results as the combined population, while the female tests were insignificant.
