# Supplementary material for: Image-based metric of invasiveness predicts response to adjuvant temozolomide for primary glioblastoma
Source: PLoS One. 2020 Mar 27;15(3):e0230492. doi: 10.1371/journal.pone.0230492 (PMC7100932; doi:10.1371/journal.pone.0230492)
Supplement: S12 Fig — Male responders (n = 28) had better overall survival than male non-responders (n = 32), while females did not have a significant survival difference between responders (n = 17) and non-responders (n = 13). Neither males nor females had a significant difference in progression free survival between responders (male n = 15, female n = 1) and non-responders (male n = 17, female n = 2). (DOCX) [file pone.0230492.s012.docx]

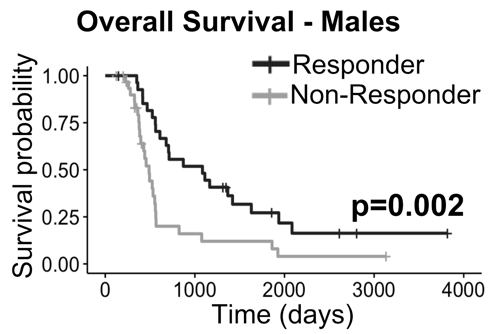

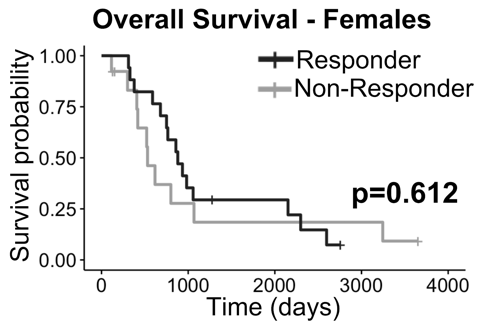

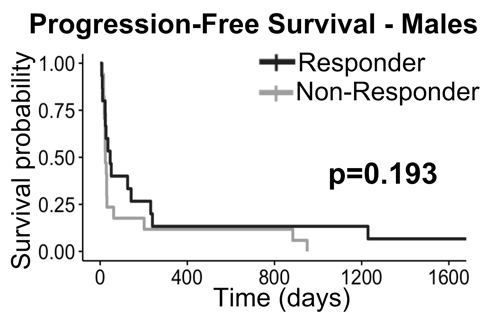

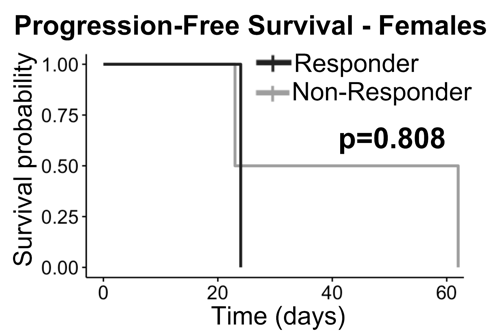


**Supplemental Figure S12. Figure 3 split into males and females.** Male responders (n=28) had better overall survival than male non-responders (n=32), while females did not have a significant survival difference between responders (n=17) and non-responders (n=13). Neither males nor females had a significant difference in progression free survival between responders (male n=15, female n=1) and non-responders (male n=17, female n=2).
