# Supplementary material for: Image-based metric of invasiveness predicts response to adjuvant temozolomide for primary glioblastoma
Source: PLoS One. 2020 Mar 27;15(3):e0230492. doi: 10.1371/journal.pone.0230492 (PMC7100932; doi:10.1371/journal.pone.0230492)
Supplement: S13 Fig — Similar to the combined population, males with nodular tumors (n = 20) have a significantly negative trend between cycles of TMZ and volumetric change and this volumetric change results in a survival difference, while neither of these observations are significant among diffuse male tumors (n = 20). Visually, females show similar trends as the combined population, but the small female population size (nodular n = 10, diffuse n = 10) likely contributes to the statistical insignificance of these observations. (DOCX) [file pone.0230492.s013.docx]

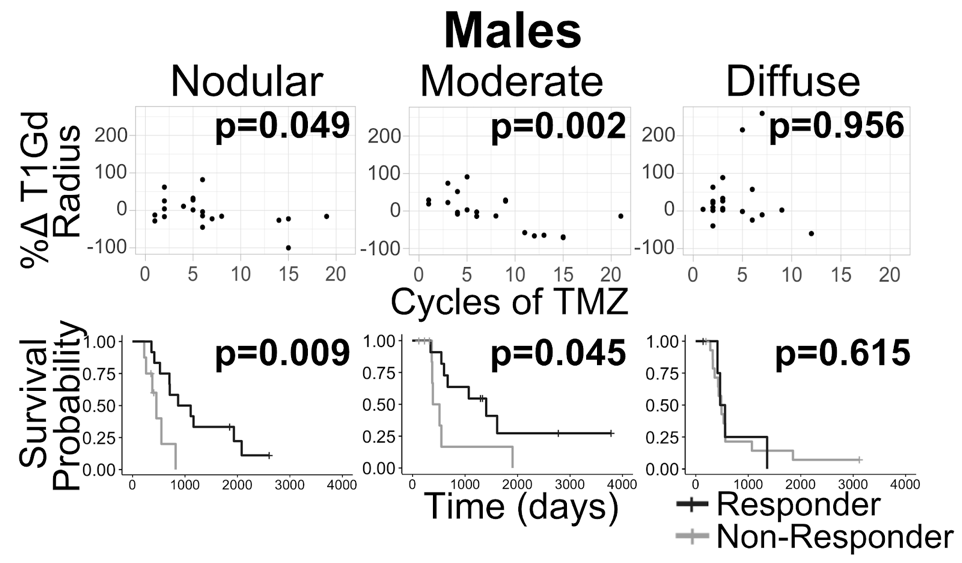


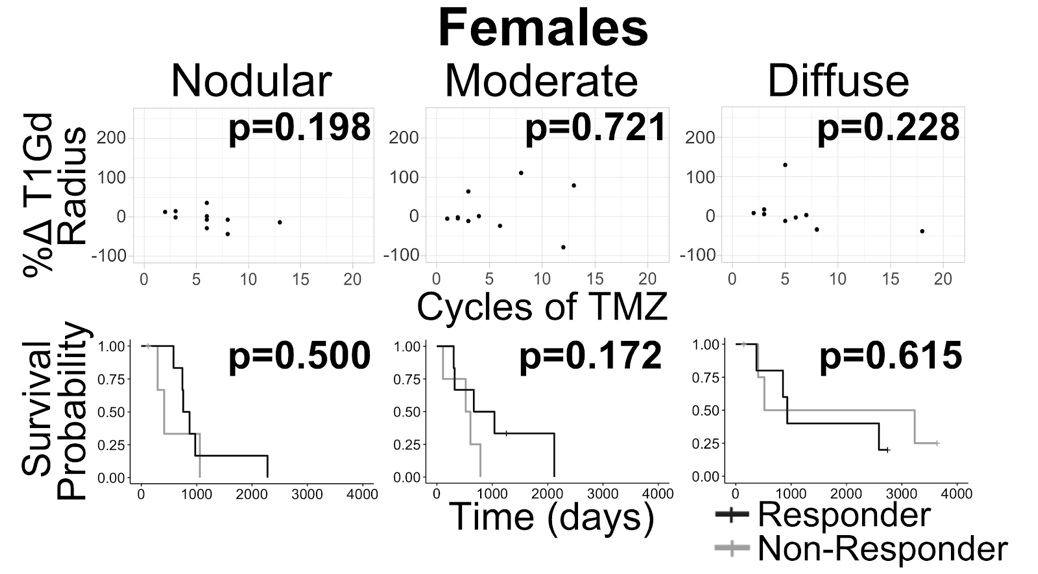


**Supplemental Figure S13. Figure 4 split into males and females.** Similar to the combined population, males with nodular tumors (n=20) have a significantly negative trend between cycles of TMZ and volumetric change and this volumetric change results in a survival difference, while neither of these observations are significant among diffuse male tumors (n=20). Visually, females show similar trends as the combined population, but the small female population size (nodular n=10, diffuse n=10) likely contributes to the statistical insignificance of these observations.
