# Supplementary material for: Image-based metric of invasiveness predicts response to adjuvant temozolomide for primary glioblastoma
Source: PLoS One. 2020 Mar 27;15(3):e0230492. doi: 10.1371/journal.pone.0230492 (PMC7100932; doi:10.1371/journal.pone.0230492)
Supplement: S14 Fig — Males (unmethylated n = 12, methylated n = 7) show similar trends between methylation status, volumetric change, diffusivity, and cycles of TMZ as the larger population, while there are not enough females with methylation status available (unmethylated n = 2, methylated n = 2) to draw a conclusion. (DOCX) [file pone.0230492.s014.docx]

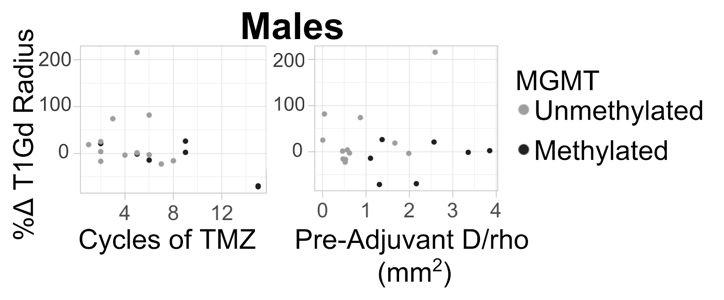

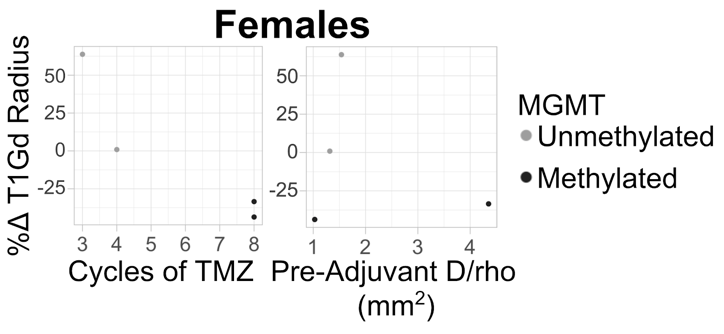


**Supplemental Figure 14.** Figure 5 split into males and females. Males (unmethylated n=12, methylated n=7) show similar trends between methylation status, volumetric change, diffusivity, and cycles of TMZ as the larger population, while there are not enough females with methylation status available (unmethylated n=2, methylated n= 2) to draw a conclusion.
