# Supplementary material for: Image-based metric of invasiveness predicts response to adjuvant temozolomide for primary glioblastoma
Source: PLoS One. 2020 Mar 27;15(3):e0230492. doi: 10.1371/journal.pone.0230492 (PMC7100932; doi:10.1371/journal.pone.0230492)
Supplement: S1 Table — Nadir is defined as the lowest volume on T1Gd imaging (converted to spherically equivalent radii for analysis) after the pre-adjuvant date and on or before the post-adjuvant date. bFor example, a value of 1 means that nadir occurred on the post-adjuvant image date, 100% of the way through adjuvant therapy. A value of 0.5, means that nadir occurred halfway through adjuvant therapy. (DOCX) [file pone.0230492.s015.docx]

**Nadir Analysis**

|  | N= | Mean | Median | Range |
| --- | --- | --- | --- | --- |
| Radii at Nadir (T1Gd) (mm) | 90 | 10.34 | 9.645 | 0-22.22 |
| %ΔT1Gd-Nadir  (From pre-adjuvant to nadir) | 90 | -11.19 | -11.86 | -100 - 215.7 |
| Time to nadir  (Days from pre-adjuvant date) | 90 | 124.1 | 69 | 20-512 |
| Proportion of time through adjuvant that nadir occurred^b^ | 90 | 0.7842 | 1 | 0.1386- 1 |
| Nadir timing  Nadir before post-adjuvant date  Nadir at post-adjuvant date | 41  (45.6%)  49  (54.4%) | -----  ----- | -----  ----- | -----  ----- |

**Supplemental Table S1. Distributions and counts of variables related to nadir analysis.** Nadir is defined as the lowest volume on T1Gd imaging (converted to spherically equivalent radii for analysis) after the pre-adjuvant date and on or before the post-adjuvant date.

^b^For example, a value of 1 means that nadir occurred on the post-adjuvant image date, 100% of the way through adjuvant therapy. A value of 0.5, means that nadir occurred halfway through adjuvant therapy.
